# Preferences of Patients With Tuberculosis for AI-Assisted Remote Health Management: Discrete Choice Experiment

**DOI:** 10.2196/77491

**Published:** 2025-09-26

**Authors:** Luo Xu, Qian Fu, Xiaojun Wang

**Affiliations:** 1 School of Medicine and Health Management, Tongji Medical College, Huazhong University of Science and Technology Wuhan China; 2 Wuhan Pulmonary Hospital, Medical Department, Jianghan University Wuhan China

**Keywords:** tuberculosis, artificial intelligence, AI, remote health management, patient preference, discrete choice experiment

## Abstract

**Background:**

Tuberculosis remains a major global public health challenge, especially in low-resource settings where long-term treatment adherence and regular follow-up are critical. The integration of artificial intelligence (AI) into remote health management has the potential to improve care delivery and patient outcomes. However, evidence on the preferences of patients with tuberculosis regarding AI-assisted services remains limited.

**Objective:**

This study aimed to examine the preferences of patients with tuberculosis for AI-assisted remote health management services in China, identifying key service characteristics that influence their choices.

**Methods:**

A discrete choice experiment was conducted among 203 patients with tuberculosis in Hubei province, China. Attributes and levels were identified through a systematic literature review, qualitative interviews, and expert panel consultations. The final design included 6 attributes: interaction method, service provider, service frequency, service content, out-of-pocket cost, and service integration. Each participant completed 8 choice tasks comparing hypothetical service options constructed based on these attributes. Preferences were analyzed using a mixed logit model to account for preference heterogeneity. Additional subgroup analyses were performed to explore variations in preferences across sociodemographic characteristics.

**Results:**

All 6 attributes significantly influenced patients’ preferences (all *P* values <.05). Participants strongly favored services involving physician oversight (*P*<.001), video-based interactions (*P*<.001), and comprehensive content (*P*<.001), while higher costs were associated with lower acceptance (*P*<.001). Subgroup analyses indicated that higher-income patients demonstrated both a greater willingness to pay and a stronger preference for physician involvement. Female participants expressed a lower preference for AI-assisted physician-led services compared to AI-only configurations. Patients with higher educational attainment also reported lower preferences for physician-involved services. Age-related differences were not statistically significant. Across all subgroups, cost remained a critical determinant of service acceptance.

**Conclusions:**

Patients with tuberculosis expressed a clear preference for high-quality, human-integrated remote health management services, emphasizing the importance of physician involvement and personalized, interactive care. These findings suggest that fully AI-driven models may face resistance and that hybrid models combining AI efficiency with professional oversight are more acceptable. Policymakers and service designers should prioritize affordability, provide targeted financial support for populations considered vulnerable, and invest in digital literacy initiatives to enhance equitable access. This study provides critical evidence to support the development of AI-assisted tuberculosis management strategies that align with patient preferences and improve treatment adherence in low-resource settings.

## Introduction

### Background

Tuberculosis remains a critical global health challenge, straining public health systems and hindering economic development [1,2]. Despite significant progress in the prevention, diagnosis, and treatment of tuberculosis, the disease continues to affect millions of people, particularly in low- and middle-income countries where health infrastructure is often limited [3]. The persistence of tuberculosis, compounded by the emergence of drug-resistant strains, further complicates efforts to control its spread [4]. As such, there is an urgent need for innovative approaches to improve prevention, increase access to treatment, and strengthen the global response to tuberculosis [5].

Given the chronic and infectious nature of tuberculosis, effective disease control often depends on long-term home-based treatment, consistent follow-up, and continuous monitoring of drug adherence [6]. In this context, remote health management services have become an increasingly viable strategy to mitigate the geographic, economic, and systemic challenges that limit timely access to care, particularly for patients living in rural or underserved areas [7-9]. These services facilitate ongoing communication and data exchange between patients and health care providers outside of traditional clinical encounters, thereby supporting sustained treatment engagement, reinforcing adherence behaviors, and reducing the likelihood of treatment discontinuation [10].

More recently, advances in artificial intelligence (AI) have greatly enhanced the potential of these remote care models [11]. AI technologies enable more accurate, responsive, and scalable service delivery through features such as automated symptom monitoring, intelligent medication reminders, and personalized health education [12]. These innovations support proactive disease management and improve patient engagement, especially in long-term treatment settings [13]. In the context of tuberculosis, where prolonged adherence, home-based care, and timely clinical feedback are essential, AI-assisted remote health management services hold great promise [14,15]. By providing tailored support and overcoming persistent access barriers, AI integration can strengthen patient engagement, reduce loss to follow-up, and ultimately improve treatment outcomes [16].

However, the successful implementation of AI-assisted remote health management services depends not only on technological capability but also on alignment with patient needs and preferences [17]. In many low-resource settings, health care innovations are often designed based on clinical priorities or system-level efficiency, with limited attention to how patients perceive and interact with these services [18,19]. This mismatch between service design and user expectations can hinder patient participation and reduce the overall effectiveness of interventions [20]. For patients with tuberculosis, who often face social stigma, financial hardship, and limited digital literacy, understanding their preferences is essential to ensure the uptake and long-term sustainability of AI-enabled care [21]. To date, little empirical research has systematically examined how patients with tuberculosis evaluate and prioritize different features of AI-assisted remote health management services. Without a clear understanding of patient preferences, it is difficult to design interventions that are both acceptable and effective in real-world settings [22].

Discrete choice experiments (DCEs) offer a robust approach to quantifying individual preferences by presenting respondents with hypothetical service alternatives and analyzing their trade-offs between competing attributes [23]. This method has been widely used in health services research to inform patient-centered care design, particularly in areas involving technology-based delivery models [24,25].

### Objectives

Building on this approach, this study uses a DCE to elicit the preferences of patients with tuberculosis for key attributes of AI-assisted remote health management services. Specifically, it aims to (1) identify the most valued service features from the patients’ perspective, (2) estimate the relative importance of different service attributes and their levels, and (3) explore preference heterogeneity across sociodemographic and treatment-related subgroups. The results of this study are expected to inform the design and implementation of more acceptable, effective, and equitable AI-integrated care models for tuberculosis management.

## Methods

### Survey Design

The development of the DCE was guided by best practice recommendations for attribute and level selection in health preference research [26]. We first conducted an extensive literature review using databases such as PubMed, Embase, China National Knowledge Infrastructure, and Web of Science to identify key service characteristics relevant to AI-assisted remote health management and to understand factors influencing patient uptake of such services. To ensure contextual validity, this process was followed by 3 focus group discussions and a series of one-on-one interviews with patients with tuberculosis and health care professionals. On the basis of this triangulated input, we developed a preliminary list of candidate attributes and levels. Detailed procedures and focus group discussion findings are provided in Multimedia Appendix 1.

Subsequently, 2 rounds of expert consultation were conducted to refine and prioritize the attributes. In the first round, 9 experts in public health, respiratory medicine, digital health, and health economics independently assessed the importance of each attribute and evaluated the appropriateness of the proposed levels. On the basis of their feedback, the attributes of personalization and privacy were excluded: personalization was viewed as inherently embedded within the definition of service content, while privacy was considered a fundamental and nonnegotiable requirement in health care services. In the second round, the revised list of attributes, along with their corresponding levels, was reviewed by the same panel to ensure consensus. This iterative process resulted in the selection of 6 final attributes, which aligns with best practice recommendations for minimizing cognitive burden in DCE design [27]: interaction method, service provider, service frequency, service content, out-of-pocket cost, and service integration. Each attribute was assigned 2 or 3 levels, informed by empirical findings, expert judgment, and contextual feasibility. The final attributes and levels are presented in Table 1. Monetary values are presented in CNY ¥, converted on the basis of purchasing power parity in 2025 (CNY ¥3.71=approximately US $1).

**Table 1:** Attributes and levels used in the discrete choice experiment survey.

Potential attributes and possible levels		Description
**Interaction method**		
	Text and images	Interaction between the service provider and patient through text or images
	Voice	Communication between the patient and service provider through voice
	Video	Bidirectional interaction through video
**Service provider**		
	AI^a^	Health management services primarily driven by AI, with health care providers intervening only when necessary or in key decisions
	AI+physician	AI provides most health management services; health care providers confirm results and provide guidance and support based on patient feedback
	Physician	Health care decisions and care coordination remain under health care providers; AI systems operate exclusively in an assistive capacity (as a tool for analyzing patient data)
**Service frequency**		
	Weekly	Health management service provided once a week
	Biweekly	Health management service provided once every 2 weeks
	Monthly	Health management service provided once a month
**Service content**		
	Basic	Includes medication reminders, adherence monitoring, and basic physiological metrics such as cough frequency and temperature
	Enhanced	Builds on basic services by adding lifestyle guidance (eg, diet and sleep) and health education
	Comprehensive	Combines basic and enhanced services with psychological support, emotional assessments, and personalized rehabilitation recommendations
**Out-of-pocket cost**		
	CNY ¥0	Fully covered by government or health care institutions, no cost to the patient
	CNY ¥25	Patient pays CNY ¥25 per month, with the remainder covered by insurance or health care institution
	CNY ¥50	Patient pays CNY ¥50 per month, with the remainder covered by insurance or health care institution
**Service integration**		
	Partial	Remote services are only partially integrated with the patient’s health care institution (eg, periodic health report or notifications regarding data outside the reference range), and health care providers need to actively query data and coordinate the treatment process
	Full	Remote services are seamlessly integrated with the offline health care institution, with health data synchronized in real time; health care providers can directly view updated data for clinical decision-making, and patients do not need to repeat information

^a^AI: artificial intelligence.

### Questionnaire and Choice Sets

The questionnaire was divided into 3 main sections: an introduction, a general information section, and the DCE choice tasks. The introduction explained the purpose of the study and provided clear instructions on how to complete the questionnaire. The general information section collected sociodemographic data, including age, gender, educational level, and medical history, to identify factors that may influence the preferences of patients with tuberculosis for AI-based remote health management services.

To construct the choice sets, we adopted an unlabeled design to ensure that respondents focused equally on all attribute dimensions [28]. Although a full factorial design combining all possible levels would ideally be used, this approach would have generated 486 choice sets (3^5^ × 2), resulting in an excessive cognitive burden for participants. Presenting such a large number of scenarios would not only cause respondent fatigue but also increase the likelihood of biased responses and require considerable time and resources [29]. To address these challenges while preserving statistical efficiency, we used a D-efficient design using Stata 16 (StataCorp LLC). This process yielded 16 choice sets, which were randomly divided into 2 blocks, with each respondent assigned to one of the blocks and presented with 8 tasks. To assess the internal consistency of participants’ responses, the second choice set in each block was repeated as the ninth task (not included in the data analysis).

Each task presented participants with 2 unlabeled service alternatives, and no opt-out option was included to focus exclusively on respondents’ relative preferences across service profiles. Attribute combinations were generated under partial constraints to ensure realism and feasibility. Before formal data collection, a pilot study was conducted with 18 patients with tuberculosis to assess the clarity, cognitive load, and interpretability of the survey instrument. On the basis of participant feedback, minor revisions were made to improve the wording of attribute descriptions and to incorporate visual aids to enhance comprehension (a visual illustration of the AI-assisted remote tuberculosis management platform). An example of the final choice set is shown in Table 2.

**Table 2:** Example choice set presented in the discrete choice experiment survey.

Attribute	Service option 1	Service option 2
Interaction method	Video	Text and images
Service provider	Physician	AI^a^+physician
Service frequency	Biweekly	Weekly
Service content	Enhanced services	Comprehensive services
Service integration	Full integration	Partial integration
Out-of-pocket cost	CNY ¥25 per month	CNY ¥50 per month
Your chosen option	—^b^	—

^a^AI: artificial intelligence.

^b^Blank cells are for participants to enter their options.

### Survey Sample

This study targeted individuals diagnosed with tuberculosis, including both patients currently undergoing treatment and those who had completed treatment. Inclusion criteria were (1) a confirmed diagnosis of pulmonary tuberculosis, (2) the ability to read and understand written text, and (3) the capacity to independently complete the questionnaire. Individuals with severe cognitive impairment or other health conditions that would interfere with survey participation were excluded. On the basis of the commonly used rule of thumb for DCEs, the minimum required sample size was estimated to be 160 participants, taking into account the number of choice sets, alternatives, attribute levels, and an anticipated proportion of invalid responses [30].

Participant recruitment was organized by the Wuhan Tuberculosis Prevention and Control Management Agency. Eligible patients with tuberculosis were recruited from multiple cities in Hubei province, including Wuhan, Jingzhou, and surrounding districts and rural areas. Participants were identified through local health facilities and registries of patients with tuberculosis. Trained staff administered the questionnaire through face-to-face interviews, with the option of electronic completion via WeChat or email if preferred.

### Data Analysis

All questionnaire responses were double entered into EpiData 3.1 software (EpiData Association) to ensure data accuracy and then imported into Stata 16 for statistical analysis. Descriptive statistics were used to summarize participants’ sociodemographic characteristics, as well as their awareness and attitudes toward telehealth and AI, to provide context for potential preference heterogeneity. A mixed logit model was used to examine the preferences of patients with tuberculosis for AI-assisted remote health management services. This model is suitable for analyzing repeated choice data and accounts for unobserved heterogeneity by allowing for random variation in preferences across individuals [31].

Attribute levels were dummy coded, with the exception of out-of-pocket cost, which was treated as a continuous variable. Willingness to pay (WTP) for improvements in each attribute level was estimated by taking the ratio of the attribute coefficient to the negative coefficient of the cost attribute. The relative importance of each attribute was derived by calculating the range between its highest and lowest estimated coefficients and expressing this range as a proportion of the sum of the ranges across all attributes. This approach quantifies the extent to which each attribute contributed to respondents’ choices, allowing direct comparison of their influence. Scenario-based choice probability simulations were not included in the final analysis, as the combination of relative importance and WTP estimates already provided comprehensive insights into patients’ trade-offs and preferences. Moreover, the absence of an opt-out option in the design limited the interpretability of simulated uptake probabilities.

Subgroup analyses were performed by stratifying participants according to age, gender, educational level, and income, allowing for the exploration of preference variations across different demographic groups. To facilitate interpretation and model convergence, selected demographic variables were recoded into binary indicators for interaction analyses based on theoretical relevance and sample distribution. All statistical tests were 2-tailed, with a significance threshold of *P*<.05.

### Ethical Considerations

This study was approved by the ethics committee of Wuhan Pulmonary Hospital (20241218) and conducted in accordance with the Declaration of Helsinki. Written informed consent was obtained from all participants before enrollment. Participants were assured of confidentiality, and all data were collected anonymously and stored securely; no personally identifiable information was collected or used. No compensation was provided for participation. No images identifying individual participants are included in the manuscript or multimedia appendices.

## Results

### Study Populations

A total of 236 individuals diagnosed with tuberculosis were recruited between February and April 2025 in Hubei province, China. After data quality and consistency checks, 32 (13.6%) of the 236 questionnaires were excluded due to incompleteness or patterned responses, leaving 203 (86%) valid responses for the final analysis.

The demographic and clinical characteristics of the participants are summarized in Multimedia Appendix 2. More than half of the participants were male (116/203, 57.1%) and aged less than 40 years (107/203, 52.7%). Approximately 30.1% (61/203) held a college degree or above, while 32.5% (66/203) had completed only junior high school or less. Most of the participants (175/203, 86.2%) reported a monthly household income below CNY ¥10,000, and 70% (142/203) resided in urban areas. Regarding employment, 55.2% (112/203) were employed, while 39.9% (81/203) were not. Nearly all participants (193/203, 95.1%) had national health insurance, although only 25.1% (51/203) had supplementary coverage. In terms of health care access, 88.7% (180/203) could reach a medical provider within 30 minutes. Clinically, 65% (132/203) were undergoing tuberculosis treatment, with 94.1% (191/203) being newly diagnosed cases. While 59.1% (120/203) reported regular in-person follow-up as their primary method of disease management, 38.9% (79/203) relied on self-management. Telemedicine use remained low: 41.4% (84/203) had never used remote health services, and only 14.8% (30/203) used them at least once a month. Most of the participants (144/203, 70.9%) reported receiving health management services once a month or less frequently.

### Preference Analyses for Mixed Logit Models

Table 3 shows the results of the mixed logit model estimating the preferences of patients with tuberculosis for AI-based remote health management services. All 6 attributes were found to significantly influence participants’ choices, indicating that the design dimensions of remote service delivery are important to patients with tuberculosis. However, not all attribute levels were statistically significant compared to their respective reference categories.

**Table 3:** Mixed logit estimates of the preferences of patients with tuberculosis for artificial intelligence (AI)–assisted services (n=203)^a^.

Attribute (reference) and attribute levels			Coefficient (SE; 95% CI)		SD (SE; 95% CI)
**Cost**					
	N/A^b^	−0.0315 (0.0046; −0.0406 to −0.0224)		0.0482 (0.0056; 0.0374 to 0.0591)	
**Interaction method (text and images)**					
	Voice	0.2776 (0.1191; 0.0441 to 0.5110)		−0.1361 (0.2037; −0.5354 to 0.2632)	
	Video	0.5548 (0.1181; 0.3232 to 0.7863)		0.4170 (0.1901; 0.0445 to 0.7896)	
**Service provider (AI)**					
	AI+physician	0.7248 (0.1395; 0.4513 to 0.9983)		−0.3493 (0.2923; −0.9221 to 0.2236)	
	Physician	0.8326 (0.1385; 0.5611 to 1.1041)		0.6762 (0.1883; 0.3072 to 1.0452)	
**Service frequency (weekly)**					
	Biweekly	0.3442 (0.1325; 0.0846 to 0.6038)		0.1184 (0.1495; −0.1746 to 0.4115)	
	Monthly	0.0632 (0.1707; −0.2714 to 0.3978)		0.0559 (0.2121; −0.3597 to 0.4716)	
**Service content (basic)**					
	Enhanced	0.2890 (0.0993; 0.0943 to 0.4837)		0.0541 (0.1887; −0.3157 to 0.4239)	
	Comprehensive	0.5237 (0.1332; 0.2627 to 0.7848)		−0.7111 (0.1753; −1.0546 to −0.3676)	
**Service integration (partial)**					
	Full	0.2151 (0.1023; 0.0145 to 0.4156)		−0.5240 (0.1559; −0.8296 to −0.2185)	

^a^Log-likelihood=−925.634; likelihood ratio test: *χ*²_10_=122.8; *P*<.001.

^b^N/A: not applicable.

Among interaction methods, participants showed stronger preferences for voice (coefficient=0.2776, 95% CI 0.0441-0.5110; *P*=.02) and especially video communication (coefficient=0.5548, 95% CI 0.3232-0.7863; *P*<.001) over text and image formats. For service providers, AI combined with physicians (coefficient=0.7248; 95% CI 0.4513-0.9983; *P*<.001) and human physicians alone (coefficient=0.8326, 95% CI 0.5611-1.1041; *P*<.001) were both significantly preferred over AI-only services. In terms of service frequency, fortnightly follow-up was significantly preferred over weekly follow-up (coefficient=0.3442, 95% CI 0.0846-0.6038; *P*=.009), while monthly follow-up showed no significant effect. Comprehensive service content (coefficient=0.5237, 95% CI 0.2627-0.7848; *P*<.001) and enhanced service content (coefficient=0.2890, 95% CI 0.0943-0.4837; *P*=.004) were preferred over basic service content. Full integration of services was also rated positively (coefficient=0.2151, 95% CI 0.0145-0.4156; *P*=.04). As expected, higher out-of-pocket costs significantly reduced the likelihood of service use (coefficient=−0.0315, 95% CI −0.0406 to −0.0224; *P*<.001). Several significant SDs were observed for attributes such as video interaction, physician-only provision, and comprehensive service content, indicating substantial heterogeneity in preferences among respondents. While most of the participants generally favored these features, the intensity of preference varied notably across individuals. This underscores the importance of designing flexible service models that can accommodate diverse patient needs and enhance overall acceptance.

The relative importance of each attribute in shaping the preferences of patients with tuberculosis for AI-assisted remote health management services was calculated at the aggregate level using the range of mean coefficients from the mixed logit model. These aggregate values do not account for individual-level heterogeneity captured by the random parameters. Out-of-pocket cost emerged as the most influential attribute, followed by service provider, interaction method, and service frequency, while service content and service integration were considered less important (Table 4).

**Table 4:** Relative importance of service attributes in influencing the preferences of patients with tuberculosis.

Attribute	Relative importance (%)
Out-of-pocket cost	38.6
Service provider	20.6
Interaction method	14
Service frequency	12.5
Service content	8.8
Service integration	5.2

### WTP Analysis

Table 5 summarizes the mean WTP values and their corresponding 95% CIs, illustrating the amounts that patients with tuberculosis would be willing to pay to obtain preferred service characteristics compared to the reference levels. These WTP estimates are based on the linear cost assumption, the validity of which was evaluated through a likelihood ratio test (Multimedia Appendix 3). The test results indicated no statistically significant difference between the linear and categorical cost specifications, suggesting that the relationship between cost and utility is adequately represented by a linear form within the examined range. Consistent with this, the estimated cost coefficients displayed an approximately proportional pattern, with the coefficient for CNY ¥50 being close to twice that for CNY ¥25, supporting the appropriateness of this assumption for WTP calculation.

**Table 5:** Estimates of willingness to pay (WTP) for service attribute levels (CNY ¥3.71=approximately US $1).

Attribute (reference) and attribute levels			WTP (CNY ¥; 95% CI)		*P* value
**Interaction method (text and images)**					
	Voice	8.81 (1.16 to 16.47)		.02	
	Video	17.61 (8.47 to 26.75)		<.001	
**Service provider (AI^a^)**					
	AI+physician	23.01 (13.11 to 32.91)		<.001	
	Physician	<.001 (15.81 to 37.05)		<.001	
**Service frequency (weekly)**					
	Biweekly	10.93 (2.35 to 19.50)		.01	
	Monthly	2.01 (−8.57 to 12.58)		.71	
**Service content (basic)**					
	Enhanced	9.17 (2.63 to 15.72)		.006	
	Comprehensive	16.63 (7.40 to 25.85)		<.001	
**Service integration (partial)**					
	Full	6.83 (−0.12 to 13.78)		.05	

^a^AI: artificial intelligence.

The results show that participants placed the highest monetary value on service provider–related attributes. Respondents were willing to pay CNY ¥26.43 (95% CI CNY ¥15.81-CNY ¥37.05) for follow-up care provided by a human physician rather than AI alone and CNY ¥23.01 (95% CI CNY ¥13.11-CNY ¥32.91) for a combination of AI and physician. In terms of the interaction method, patients were willing to pay CNY ¥17.61 (95% CI CNY ¥8.47-CNY ¥26.75) to switch from text- and image-based services to video communication and CNY ¥8.81 (95% CI CNY ¥1.16-CNY ¥16.47) for voice-based interactions. Respondents also valued improvements in service content and frequency. They were willing to pay CNY ¥16.63 (95% CI CNY ¥7.40-CNY ¥25.85) for comprehensive services and CNY ¥9.17 (95% CI CNY ¥2.63-CNY ¥15.72) for enhanced services compared to basic services. Fortnightly service frequency was associated with a WTP value of CNY ¥10.93 (95% CI CNY ¥2.35-CNY ¥19.50), whereas monthly service frequency did not yield a statistically significant WTP premium (*P*=.71).

Finally, service integration was also valued: participants were willing to pay CNY ¥6.83 (95% CI –CNY ¥0.12 to CNY ¥13.78) to move from partial to full integration, although this estimate was marginally significant (*P*=.05). Overall, these findings highlight a strong preference among patients with tuberculosis for humanized, high-quality, and accessible remote management services, while emphasizing the importance of careful interpretation of WTP values within the constraints of the cost structure.

### Interaction Effects Analysis

To further explore preference heterogeneity among different patient groups, we included interaction terms between key sociodemographic characteristics and service attributes in a comprehensive mixed logit model. Detailed estimates for all interactions are presented in Multimedia Appendix 4.

The analysis identified several statistically significant patterns. Participants with higher income levels exhibited significantly lower price sensitivity compared to those with lower incomes, as indicated by a positive interaction between income and cost (β=.673; *P*=.009). This suggests that, for higher-income individuals, increases in out-of-pocket cost have a smaller negative impact on utility. Female respondents demonstrated a significantly lower preference for AI-assisted physician-led services compared to AI-only configurations (β=−.647; *P*=.003). Similarly, participants with higher educational attainment expressed lower preferences for AI-assisted physician-led services (β=−.634; *P*=.02). By contrast, higher-income participants showed a stronger preference for AI-assisted physician-led services (β=.673; *P*=.009), indicating that income influences both price sensitivity and service provider configuration preferences. No other cost-related interactions reached statistical significance, although the interaction with gender approached significance (*P*=.09). No statistically significant interactions were observed for age or for other sociodemographic variables across the remaining attributes.

Overall, these findings highlight the significant roles of income, education, and gender in shaping service preferences and, in the case of income, price sensitivity. The results emphasize the importance of accounting for demographic heterogeneity, particularly differences related to income level, educational attainment, and gender, when designing and pricing AI-assisted remote tuberculosis management services.

## Discussion

### Principal Findings

To our knowledge, this study is the first to examine the preferences of patients with tuberculosis for AI-assisted remote health management services in China using a DCE. tuberculosis requires long-term treatment, carries a high transmission risk, and demands intensive public health surveillance [32], making it especially suitable for AI integration in resource-constrained settings. By quantifying how patients with tuberculosis prioritize different service attributes, this study fills an important gap and provides evidence to guide patient-centered service design.

All 6 attributes significantly influenced patients’ preferences, with cost having the greatest impact. Even modest increases in cost (from CNY ¥0 to CNY ¥25 or CNY ¥50) markedly reduced service uptake, emphasizing that affordability remains a critical barrier. This finding aligns with previous DCE studies in low-income settings. Despite this cost sensitivity, patients showed strong preferences for higher service quality. Specifically, switching from text and images to voice or video interactions, replacing AI-only service providers with physician-led models, and expanding service content from basic to comprehensive all increased the likelihood of selecting a service package. These results suggest that although patients are sensitive to price, they are willing to pay more for personalized interactions, professional oversight, and comprehensive support.

### Comparison to Prior Work

Our findings are consistent with prior DCEs on follow-up care among survivors of cancer and patients with chronic diseases [33-35], which have emphasized the importance of service provider identity and the comprehensiveness of care; for example, studies in cancer follow-up have shown that patients strongly prefer care led by specialists rather than general practitioners and place high value on thorough clinical assessments and continuity with trusted health care providers. These preferences reflect a broader trend across different disease contexts, where patients prioritize professional oversight and detailed, personalized support in their ongoing care [33-35].

This study extends existing evidence by introducing AI-assisted service models into the decision-making framework for tuberculosis management. While AI technologies offer the potential for operational efficiency and greater scalability, our results indicate that patients remain cautious about fully automated services. The strong preference for hybrid models that combine AI with physician oversight and for fully physician-led services suggests that human involvement is perceived as essential for fostering trust, providing reassurance, and ensuring individualized care [36]. This preference aligns with prior research indicating that AI is more acceptable when integrated with human expertise rather than used as a stand-alone replacement [37,38].

The WTP analysis further supports this conclusion. Patients were willing to pay the highest premium, more than CNY ¥26 (approximately US $7 in purchasing power parity terms), for physician-led services and an additional CNY ¥17 (approximately US $4.50 in purchasing power parity terms) for video-based interactions. This willingness highlights a clear preference for human-centered and interactive service elements. These observations align with research suggesting that AI is most acceptable to patients when it functions as a complementary tool that supports, rather than replaces, human expertise. In chronic conditions that require long-term management, such as tuberculosis, the perceived irreplaceability of personalized medical judgment and emotional support becomes particularly evident.

Subgroup and interaction analyses revealed notable differences in preferences across sociodemographic groups, reinforcing the need to account for patient heterogeneity in service design. Female participants exhibited a significantly lower preference for AI-assisted physician-led services compared to AI-only configurations, which may reflect differing expectations regarding the added value of human involvement in AI-mediated care [39]. Similarly, participants with higher educational attainment also expressed lower preferences for AI-assisted physician-led services, potentially indicating a more critical appraisal of whether increased complexity and resource input translate into perceived benefits. By contrast, individuals with higher incomes demonstrated both a significantly lower sensitivity to cost increases and a stronger preference for AI-assisted physician-led configurations, underscoring the role of economic capacity in facilitating the acceptance of more resource-intensive service models and raising concerns about inequitable access if financial barriers remain unaddressed [40]. Although our findings did not show statistically significant age-related differences, prior studies indicate that with appropriate support and confidence-building measures, older adults can effectively engage with more interactive and integrated service models [41]. These findings suggest that certain groups, such as individuals with higher incomes, higher educational attainment, or specific gender-related preferences, may be more receptive to advanced and personalized AI-assisted services, whereas others may require additional measures to facilitate effective engagement. Policy and program design should therefore integrate both technological and socioeconomic considerations [42]. Potential strategies include providing targeted financial subsidies to mitigate cost-related barriers for populations with lower-income status and implementing tailored digital literacy and engagement initiatives to strengthen technology readiness, particularly among older adults and other groups considered vulnerable who may face greater challenges in adopting AI-enabled health care services [43].

### Limitations

Several limitations should be noted. First, although the DCE method captures stated preferences, actual behaviors in real-world settings may not entirely align due to factors such as access to digital tools or tuberculosis-related stigma. Second, the sample size was estimated using a traditional rule-of-thumb approach rather than more advanced model-based methods (eg, S-estimate), which may have implications for the precision of our estimates. Third, while we conducted a pilot test to evaluate clarity and cognitive burden, we did not explicitly label it as a formal pretest following best practice recommendations (eg, using iterative cognitive interviews and pretesting checklists), which may have limited the refinement of certain items. In addition, the exclusion of an opt-out option could have led to a slight overestimation of uptake probabilities. Finally, as the study focused on patients with tuberculosis in Hubei province, the findings may not be fully generalizable to other regions or patient groups.

### Future Directions

Future research should validate these findings in diverse geographic and demographic contexts and incorporate longitudinal designs to assess real-world adoption and sustained engagement. Policymakers should consider stratified implementation strategies, including subsidized service models for groups considered economically disadvantaged and targeted digital literacy initiatives to improve accessibility for older adults and rural residents.

### Conclusions

This study systematically examined the preferences of patients with tuberculosis for AI-assisted remote follow-up care in China. Patients placed the highest overall importance on affordability, followed by who provides the service and how the interaction takes place, indicating that both economic and quality-related considerations shape decision-making. While lower costs were generally preferred, many patients were willing to pay more for physician involvement and richer communication formats. Analysis of preference heterogeneity showed that income, education, and gender influenced the relative appeal of certain service configurations, whereas age and treatment history played a less decisive role. Together, these findings provide a comprehensive understanding of which service features matter most, how their relative influences compare, and how preferences vary across patient subgroups, offering a robust evidence base for patient-centered service design.

## Appendix

Multimedia Appendix 1 Information on discrete choice experiment development.

Multimedia Appendix 2 Population statistics.

Multimedia Appendix 3 Linearity test.

Multimedia Appendix 4 Subgroup interactions and main effects.

## Abbreviations

AI: artificial intelligence
DCE: discrete choice experiment
WTP: willingness to pay

## Data Availability

The datasets generated and analyzed during this study are available from the corresponding author on reasonable request.
