# Supplementary material for: Preferences of Patients With Tuberculosis for AI-Assisted Remote Health Management: Discrete Choice Experiment
Source: J Med Internet Res. 2025 Sep 26;27:e77491. doi: 10.2196/77491 (PMC12514403; doi:10.2196/77491)
Supplement: Multimedia Appendix 1 [file jmir_v27i1e77491_app1.docx]

Study Design

This study follows the design principle of a DCE to explore the preferences of TB patients for AI-assisted remote health management services. The DCE development process includes the following stages: identifying and defining key attributes and levels, generating choice sets, designing the questionnaire, collecting survey data, and analyzing the results. The main stages of the DCE are outlined in Figure 1.


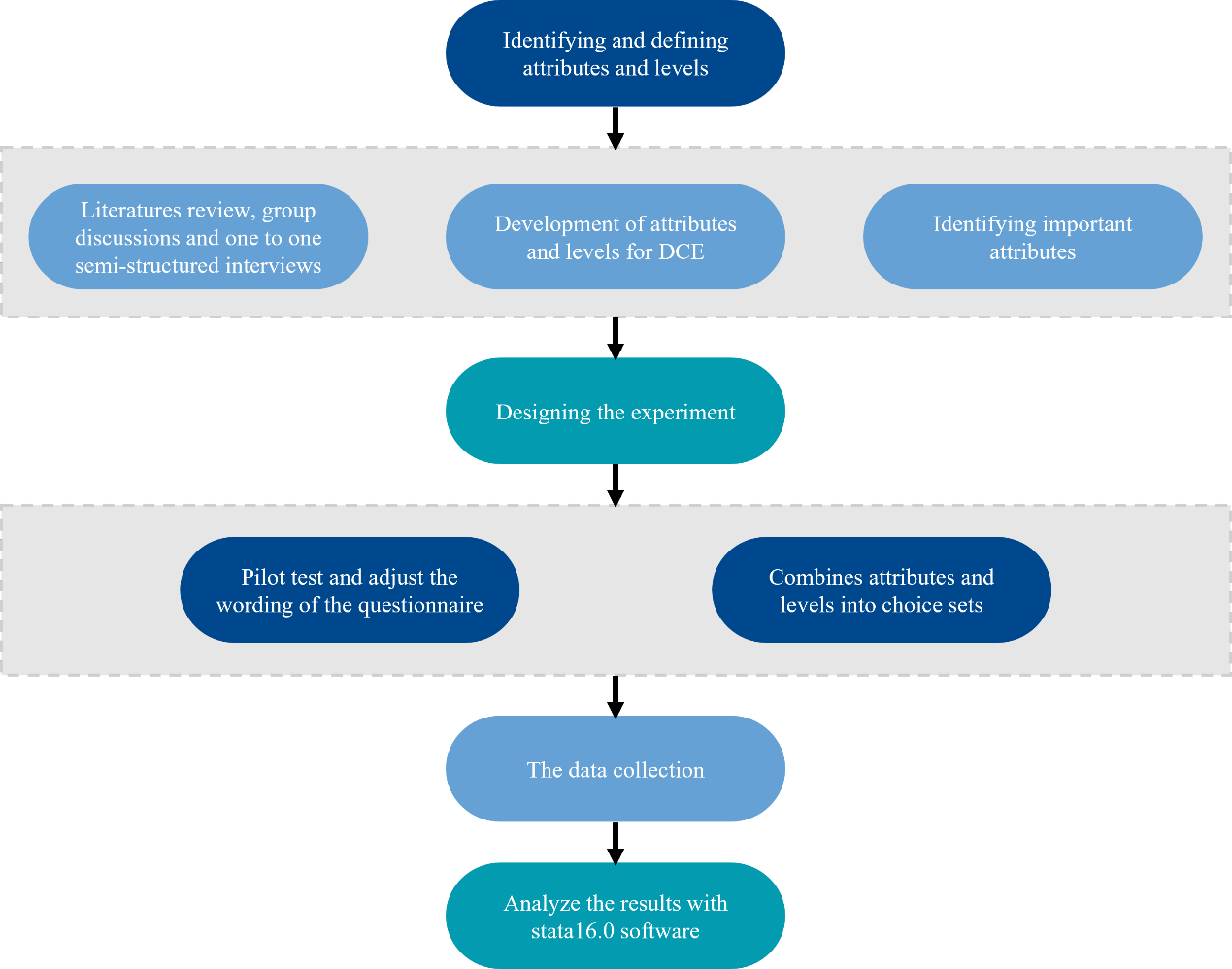


**Figure 1** The development process of the DCE.

Literature Review

An extensive literature review was conducted to identify potential attributes for inclusion in the DCE. The search encompassed PubMed, Embase, CNKI, and Web of Science, using the following search terms: (“Tuberculosis” OR “TB”) AND (“Patient Preference” OR “Patient Satisfaction”) AND (“Telehealth” OR “Health Management” OR “Artificial Intelligence”) AND (“Chronic Disease” OR “Follow-up”). In addition to focusing on TB-specific research, studies on the management of other chronic diseases requiring long-term home-based care were included to inform our understanding of patient preferences for telehealth and AI-driven interventions. To ensure comprehensive coverage, references from the retrieved studies were also reviewed, which helped to identify additional research on patient preferences, psychosocial support and follow-up strategies in chronic disease management. Furthermore, given the unique healthcare context in China, it is crucial to consider institutional factors such as the cost of healthcare services, which is an important attribute when assessing patient preferences in this setting.

FGDs and One-on-One Interviews

To refine the potential attributes identified in the literature review and gain a deeper understanding of TB patients' preferences for AI-assisted remote health management services, we conducted FGDs and one-on-one interviews. The goal was to collect both context-specific insights and expert opinions to guide the design of the DCE. Participants were selected using purposive sampling to ensure a diverse range of perspectives. Healthcare providers, including doctors, nurses, and health professionals experienced in TB care, were chosen to provide expert insights. Additionally, TB patients were recruited to reflect a variety of demographic characteristics (e.g., age, gender, education level, and disease stage). This approach ensured the inclusion of a broad spectrum of opinions, helping to capture both the healthcare providers’ and patients’ viewpoints on AI-assisted health management.

We conducted four separate FGDs, with each group comprising 5-6 participants. These sessions were designed to foster discussion and elicit a variety of opinions. In addition to the FGDs, one-on-one interviews were held with three experts: a TB specialist, a head nurse with expertise in chronic disease management, and a health economist.

**Preliminary list of potential attributes and levels**

| **Potential Attributes** | **Possible Levels** | **Definition** |
| --- | --- | --- |
| **Interaction Method**^[1]–[7]^ | Text and Image | Interaction between the service provider and patient through text or image forms. |
|  | Voice | Communication between the patient and service provider through voice. |
|  | Video | Bidirectional interaction through video, allowing healthcare providers or AI to assess patient signs, emotions, and overall status. |
| **Service Provider**^[2],[8]–[12]^ | AI | Health management services are primarily driven by AI, with healthcare providers only intervening when necessary or in key decisions. |
|  | AI + Doctor | AI provides most of the health management services, while healthcare providers confirm results and provide guidance and support based on patient feedback. |
|  | Doctor | Health management is fully performed by healthcare providers, with AI serving only as an auxiliary tool for analyzing patient data. |
| **Service Frequency**^[2],[5],[13]–[17]^ | Weekly | Health management service is provided once a week. |
|  | Bi-weekly | Health management service is provided once every two weeks. |
|  | Monthly | Health management service is provided once a month. |
| **Service Content**^[5],[18]–[23]^ | Basic Services | Includes medication reminders, adherence monitoring, and basic physiological metrics such as cough frequency and temperature. |
|  | Enhanced Services | Builds on basic services by adding lifestyle guidance (e.g., diet, sleep) and health education. |
|  | Comprehensive Services | Combines basic and enhanced services with psychological support, emotional assessments, and personalized rehabilitation recommendations. |
| **Out-of-pocket Cost**^[13],[24]^ | 0 RMB | Fully covered by government or healthcare institutions, no cost to the patient. |
|  | 25 RMB | Patient pays 25 RMB per month, with the remainder covered by insurance or healthcare institution. |
|  | 50 RMB | Patient pays 50 RMB per month, with the remainder covered by insurance or healthcare institution. |
| **Service Integration**^[4],[16],[25]–[27]^ | Partial Integration | Remote services are only partially integrated with the patient's healthcare institution, e.g., periodic health report or abnormal data notifications, and healthcare providers need to actively query data and coordinate the treatment process. |
|  | Full Integration | Remote services are seamlessly integrated with the offline healthcare institution, with health data synchronized in real-time. Healthcare providers can directly view updated data for clinical decision-making, and patients do not need to repeat information. |
| **Personalization Level**^[4],[10],[17],[28]^ | Standardized | Uniform service content, including basic symptom monitoring and medication reminders, with no personalization. |
|  | Partial Personalization | Adjusts some service content based on patient history and current needs, such as specific health advice. |
|  | Highly Personalized | Completely tailored services based on the patient’s specific health condition and needs, including detailed rehabilitation guidance, lifestyle recommendations, and psychological support. |
| **Privacy**^[2],[10],[21],[29]^ | Strict | Health data and personal information are handled with strict confidentiality and privacy protection measures. |
|  | Loosely Controlled | Health data is collected and used with less stringent privacy controls, possibly involving more accessible data sharing. |

**Reference：**

[1] Mangan J M, Woodruff R S, Winston C A, et al. Recommendations for Use of Video Directly Observed Therapy During Tuberculosis Treatment - United States, 2023[J]. MMWR. Morbidity and mortality weekly report, 2023, 72(12): 313-316.

[2] Bedard B A, Younge M, Pettit P A, et al. Using Telemedicine for Tuberculosis Care Management: a Three County Inter-Municipal Approach[J]. Journal of Medical Systems, 2017, 42(1): 12.

[3] Chuck C, Robinson E, Macaraig M, et al. Enhancing management of tuberculosis treatment with video directly observed therapy in New York City[J]. The International Journal of Tuberculosis and Lung Disease: The Official Journal of the International Union Against Tuberculosis and Lung Disease, 2016, 20(5): 588-593.

[4] Kumwichar P, Prappre T, Chongsuvivatwong V. Tuberculosis Treatment Compliance Under Smartphone-Based Video-Observed Therapy Versus Community-Based Directly Observed Therapy: Cluster Randomized Controlled Trial[J]. JMIR mHealth and uHealth, 2024, 12(1): e53411.

[5] Guo P, Qiao W, Sun Y, et al. Telemedicine Technologies and Tuberculosis Management: A Randomized Controlled Trial[J]. Telemedicine and e-Health, 2020, 26(9): 1150-1156.

[6] Mayer C J, Mahal J, Geisel D, et al. User preferences and trust in hypothetical analog, digitalized and AI-based medical consultation scenarios: An online discrete choice survey[J]. Computers in Human Behavior, 2024, 161: 108419.

[7] Wang S, Shi Y, Sui M, et al. Telephone follow-up based on artificial intelligence technology among hypertension patients: Reliability study[J]. JOURNAL OF CLINICAL HYPERTENSION, 2024, 26(6): 656-664.

[8] Wijayanti E, Bachtiar A, Achadi A, et al. Mobile application development for improving medication safety in tuberculosis patients: A quasi-experimental study protocol[J]. PLOS ONE, 2022, 17(9): e0272616.

[9] Shaik T, Tao X, Higgins N, et al. Remote patient monitoring using artificial intelligence: Current state, applications, and challenges[J]. WIREs Data Mining and Knowledge Discovery, 2023, 13(2): e1485.

[10] Tinetti M E, Naik A D, Dodson J A. Moving From Disease-Centered to Patient Goals-Directed Care for Patients With Multiple Chronic Conditions: Patient Value-Based Care[J]. JAMA cardiology, 2016, 1(1): 9-10.

[11] Margineanu I, Louka C, Akkerman O, et al. eHealth in TB clinical management[J]. The International Journal of Tuberculosis and Lung Disease, 2022, 26(12): 1151-1161.

[12] Syahrul S, Irwan A M, Saleh A, et al. Effectiveness of Mobile Application–Based Intervention on Medication Adherence Among Pulmonary Tuberculosis Patients: A Systematic Review[J]. CIN: Computers, Informatics, Nursing, 2023: 10.1097/CIN.0000000000001213.

[13] Wang H, Sun H, Jin C, et al. Preference to Family Doctor Contracted Service of Patients with Chronic Disease in Urban China: A Discrete Choice Experiment[J]. Patient Preference and Adherence, 2022, 16: 2103-2114.

[14] Shalahuddin I, Pebrianti S, Eriyani T, et al. Telenursing Intervention for Pulmonary Tuberculosis Patients - A Scoping Review[J]. Journal of Multidisciplinary Healthcare, 2024, 17: 57-70.

[15] Guo N, Marra C A, FitzGerald J M, et al. Patient Preference for Latent Tuberculosis Infection Preventive Treatment: A Discrete Choice Experiment[J]. Value in Health, 2011, 14(6): 937-943.

[16] Kerkhoff A D, Chilukutu L, Nyangu S, et al. Patient Preferences for Strategies to Improve Tuberculosis Diagnostic Services in Zambia[J]. JAMA Network Open, 2022, 5(8): e2229091.

[17] Dohál M, Porvazník I, Solovič I, et al. Advancing tuberculosis management: the role of predictive, preventive, and personalized medicine[J]. Frontiers in Microbiology, 2023, 14.

[18] Lee S, Rajaguru V, Baek J S, et al. Digital Health Interventions to Enhance Tuberculosis Treatment Adherence: Scoping Review[J]. JMIR mHealth and uHealth, 2023, 11(1): e49741.

[19] Bao Y, Wang C, Xu H, et al. Effects of an mHealth Intervention for Pulmonary Tuberculosis Self-management Based on the Integrated Theory of Health Behavior Change: Randomized Controlled Trial[J]. JMIR Public Health and Surveillance, 2022, 8(7): e34277.

[20] Tello-Cajiao M E, Mosquera-Hernández J C, Ardila-Giraldo S, et al. Synchronous video-supported treatment for tuberculosis in Cali, Colombia: An implementation study[J]. Health Policy and Technology, 2023, 12(2): 100747.

[21] Kamchedzera W, Quaife M, Msukwa-Panje W, et al. Treatment preferences among people at risk of developing tuberculosis: A discrete choice experiment[J]. PLOS Global Public Health, 2024, 4(7): e0002804.

[22] Egwaga S, Range N, Lwilla F, et al. Assessment of patient preference in allocation and observation of anti-tuberculosis medication in three districts in Tanzania[J]. Patient Preference and Adherence, 2008, 2: 1-6.

[23] Yuen C M, Millones A K, Galea J T, et al. Toward patient-centered tuberculosis preventive treatment: preferences for regimens and formulations in Lima, Peru[J]. BMC Public Health, 2021, 21(1): 121.

[24] Ren Yiyang，Yin Yue，Tang Wenxi，et al. An Empirical Study on the Pricing of Primary Health Management Services：Taking Personalized Health Service Package as an Example [J]. Chinese Health Economics，2024，43（6）：5-9

[25] Rivara A C, Galárraga O, Selu M, et al. Identifying patient preferences for diabetes care: A protocol for implementing a discrete choice experiment in Samoa[J]. PLOS ONE, 2023, 18(12): e0295845.

[26] Vera Cruz G, Dlamini P S. People’s willingness and determinants to use selected tele-consultation public health services in Mozambique[J]. BMC Public Health, 2021, 21(1): 947.

[27] Milligan H, Iribarren S J, Chirico C, et al. Insights from participant engagement with the tuberculosis treatment support tools intervention: Thematic analysis of interactive messages to guide refinement to better meet end user needs[J]. International Journal of Medical Informatics, 2021, 149: 104421.

[28] Johnson K B, Wei W Q, Weeraratne D, et al. Precision Medicine, AI, and the Future of Personalized Health Care[J]. Clinical and Translational Science, 2021, 14(1): 86-93.

[29] Aschmann H E, Musinguzi A, Kadota J L, et al. Preferences of people living with HIV for features of tuberculosis preventive treatment regimens in Uganda: a discrete choice experiment[J]. Journal of the International AIDS Society, 2024, 27(12): e26390.
