# Supplementary material for: Preferences of Patients With Tuberculosis for AI-Assisted Remote Health Management: Discrete Choice Experiment
Source: J Med Internet Res. 2025 Sep 26;27:e77491. doi: 10.2196/77491 (PMC12514403; doi:10.2196/77491)
Supplement: Multimedia Appendix 2 [file jmir_v27i1e77491_app2.docx]

| characteristics | | participants (N=203） | | |
| --- | --- | --- | --- | --- |
|  |  | N | | constituent ratio (%) |
| Gender | |  | |  |
| Male | | 116 | | 57.1 |
| Female | | 87 | | 42.9 |
| Age，years  18-39  40-59 | | 107  49 | | 52.7  24.1 |
| 60-69 | | 27 | | 13.3 |
| ≥70 | | 20 | | 9.9 |
| Level of education | |  | |  |
| Junior high school and below | | 66 | | 32.5 |
| Senior high school | | 38 | | 18.7 |
| Junior college | | 38 | | 18.7 |
| College degree and above | | 61 | | 30.1 |
| Monthly household income, CNY |  | | |  |
| ＜5000 | | 98 | | 48.3 |
| 5001-10000 | | 77 | | 44.95 |
| 10001-15000 | | 12 | | 5.9 |
| ≥15000 | | 16 | | 7.9 |
| Place of residence | |  | |  |
| Urban | | 142 | | 70.0 |
| Rural | | 61 | | 30.0 |
| Employment status | |  | |  |
| Employed | | 112 | | 55.2 |
| Not employed | | 81 | | 39.9 |
| Student | | 10 | | 4.9 |
| National health insurance | |  | |  |
| Purchased | | 193 | | 95.1 |
| Not purchased | | 10 | | 4.9 |
| Supplementary health insurance  Purchased  Not purchased | | 51  152 | | 25.1  74.9 |
| Travel time to healthcare provider  Less than 15 min | | 95 | | 46.8 |
| 16-30min  30-45min  More than 45min  TB treatment status  Currently undergoing treatment  Completed treatment | | 85  17  6  132  71 | | 41.9  8.4  3.0  65.0  35.0 |
| TB patient type  New case | | 191 | | 94.1 |
| Retreatment  Daily Health Management (most commonly)  Self-management  In-person follow-up  Digital health platform | | 12  79  120  4 | | 5.9  38.9  59.1  2.0 |
| Frequency of use of telemedicine services  Never  More than semi-annually  Almost half a year.  Less than semi-annually  At least once a month  Frequency of receiving health management services  Never  More than once every two months  Once a month  Fortnightly  Once a week | | | 84  44  34  11  30  7  10  144  34  8 | 41.4  21.7  16.7  5.4  14.8  3.4  4.9  70.9  16.7  3.9 |
