# Supplementary material for: Preferences of Patients With Tuberculosis for AI-Assisted Remote Health Management: Discrete Choice Experiment
Source: J Med Internet Res. 2025 Sep 26;27:e77491. doi: 10.2196/77491 (PMC12514403; doi:10.2196/77491)
Supplement: Multimedia Appendix 3 [file jmir_v27i1e77491_app3.docx]

To evaluate whether the cost attribute can be appropriately treated as linear, two mixed logit models were estimated. The first model used dummy coding for the cost attribute, allowing each cost level to have an independent coefficient. The second model specified the cost attribute as a single continuous variable, thereby assuming a linear relationship across levels. Both models were estimated using the same analytical sample and identical random parameter specifications. A likelihood ratio (LR) test was then conducted to compare the two model specifications.

Table S2. Results of the Linearity Test for the Cost Attribute

| Model Specification | Log-Likelihood (LL) | Parameters (k) | AIC | BIC | LR Statistic | df | p-value |
| --- | --- | --- | --- | --- | --- | --- | --- |
| Dummy-coded model | -982.7594 | k₁ | AIC₁ | BIC₁ | — | — | — |
| Linear model | -983.9670 | k₂ | AIC₂ | BIC₂ | 2.415 | 1 | 0.120 |

The LR test revealed no statistically significant difference between the dummy-coded and linear specifications of the cost attribute (p = 0.120). This finding suggests that the linearity assumption cannot be rejected.
