# Supplementary material for: Preferences of Patients With Tuberculosis for AI-Assisted Remote Health Management: Discrete Choice Experiment
Source: J Med Internet Res. 2025 Sep 26;27:e77491. doi: 10.2196/77491 (PMC12514403; doi:10.2196/77491)
Supplement: Multimedia Appendix 4 [file jmir_v27i1e77491_app4.docx]

This supplementary appendix provides detailed results from the mixed logit model incorporating both the main effects of service attribute levels and their interaction terms with key sociodemographic characteristics. The purpose of including these interactions is to explore potential heterogeneity in patient preferences for AI-assisted remote health management services across different subgroups.

All service attributes were coded as dummy variables using effects coding. The reference levels for each attribute were as follows: “Text & Image” for Interaction Method, “AI” for Service Provider, “Weekly” for Service Frequency, “Basic services” for Service Content, “0 CNY” for Out-of-pocket Cost, and “Partial integration” for Service Integration. Sociodemographic moderator variables included gender (coded as 1 = female, 0 = male), age group (1 = older participants, 0 = younger participants), education level (1 = college degree or above, 0 = below college), and income level (1 = higher income, 0 = lower income).

Interaction terms were created by multiplying each attribute dummy variable by the respective moderator variable to examine whether preferences for specific service configurations differed systematically among these subgroups. Positive coefficients (either for main effects or interaction effects) indicate a stronger preference for the corresponding attribute level relative to the reference category, while negative coefficients suggest a reduced preference. Table S1 presents the estimated coefficients, standard errors, p-values, and 95% confidence intervals for both main effects and interaction terms, allowing for a nuanced interpretation of how demographic characteristics influence patient trade-offs when selecting between different service options.

Table S1 Mixed Logit Model with Interaction Effects

| **Attribute (Reference Level)** | **Attribute Level × Moderator** | **Coefficient (SE)** | **95% CI** | **p-value** | **SD (SE)** |
| --- | --- | --- | --- | --- | --- |
| **Interaction Method (Text & Image)** | Voice (main effect) | 0.187 (0.255) | -0.314, 0.687 | 0.465 | -0.008 (0.167) |
|  | Video (main effect) | 0.428 (0.251) | -0.064, 0.920 | 0.088 | 0.310 (0.194) |
| **Service Provider (AI)** | AI + Doctor (main effect) | 0.718 (0.266) | 0.196, 1.239 | 0.007** | -0.005 (0.237) |
|  | Doctor (main effect) | 0.655 (0.269) | 0.128, 1.183 | 0.015* | -0.209 (0.288) |
| **Service Frequency (Weekly)** | Bi-weekly (main effect) | 0.639 (0.275) | 0.099, 1.179 | 0.020* | -0.019 (0.145) |
|  | Monthly (main effect) | 0.465 (0.366) | -0.252, 1.182 | 0.204 | -0.046 (0.263) |
| **Service Content (Basic)** | Enhanced (main effect) | 0.317 (0.211) | -0.096, 0.729 | 0.133 | -0.019 (0.098) |
|  | Comprehensive (main effect) | 0.559 (0.251) | 0.067, 1.051 | 0.026* | -0.003 (0.183) |
| **Service Integration (Partial)** | Full (main effect) | 0.206 (0.195) | -0.177, 0.589 | 0.292 | 0.448 (0.117)*** |
| **Out-of-pocket Cost (continuous)** | Cost (main effect) | -0.028 (0.005) | -0.039, -0.017 | 0.000*** | — |
| **Interaction Method × Female** | Voice × Female | 0.257 (0.206) | -0.148, 0.661 | 0.213 | — |
|  | Video × Female | 0.375 (0.208) | -0.034, 0.783 | 0.072 | — |
| **Service Provider × Female** | AI + Doctor × Female | -0.647 (0.218) | -1.074, -0.221 | 0.003** | — |
|  | Doctor × Female | -0.207 (0.213) | -0.624, 0.210 | 0.331 | — |
| **Service Frequency × Female** | Bi-weekly × Female | -0.364 (0.225) | -0.804, 0.077 | 0.106 | — |
|  | Monthly × Female | -0.310 (0.297) | -0.893, 0.272 | 0.297 | — |
| **Service Content × Female** | Enhanced × Female | -0.027 (0.171) | -0.361, 0.308 | 0.876 | — |
|  | Comprehensive × Female | -0.372 (0.209) | -0.781, 0.037 | 0.075 | — |
| **Service Integration × Female** | Full × Female | 0.138 (0.160) | -0.176, 0.452 | 0.388 | — |
| **Cost × Female** | Cost × Female | 0.008 (0.004) | -0.001, 0.016 | 0.089 | — |
| **Interaction Method × Older Age** | Voice × Older Age | 0.141 (0.247) | -0.343, 0.626 | 0.568 | — |
|  | Video × Older Age | 0.084 (0.247) | -0.400, 0.568 | 0.734 | — |
| **Service Provider × Older Age** | AI + Doctor × Older Age | 0.360 (0.255) | -0.140, 0.860 | 0.158 | — |
|  | Doctor × Older Age | 0.446 (0.255) | -0.054, 0.947 | 0.080 | — |
| **Service Frequency × Older Age** | Bi-weekly × Older Age | -0.074 (0.267) | -0.596, 0.449 | 0.782 | — |
|  | Monthly × Older Age | -0.454 (0.353) | -1.145, 0.238 | 0.199 | — |
| **Service Content × Older Age** | Enhanced × Older Age | -0.011 (0.205) | -0.412, 0.391 | 0.959 | — |
|  | Comprehensive × Older Age | -0.148 (0.246) | -0.630, 0.333 | 0.546 | — |
| **Service Integration × Older Age** | Full × Older Age | -0.226 (0.191) | -0.601, 0.149 | 0.238 | — |
| **Cost × Older Age** | Cost × Older Age | 0.001 (0.005) | -0.010, 0.011 | 0.875 | — |
| **Interaction Method × Higher Edu** | Voice × Higher Edu | -0.009 (0.265) | -0.528, 0.510 | 0.974 | — |
|  | Video × Higher Edu | 0.187 (0.268) | -0.339, 0.712 | 0.486 | — |
| **Service Provider × Higher Edu** | AI + Doctor × Higher Edu | -0.634 (0.273) | -1.168, -0.099 | 0.020* | — |
|  | Doctor × Higher Edu | -0.514 (0.273) | -1.048, 0.021 | 0.059 | — |
| **Service Frequency × Higher Edu** | Bi-weekly × Higher Edu | -0.262 (0.285) | -0.820, 0.295 | 0.356 | — |
|  | Monthly × Higher Edu | -0.096 (0.378) | -0.838, 0.646 | 0.800 | — |
| **Service Content × Higher Edu** | Enhanced × Higher Edu | -0.112 (0.220) | -0.544, 0.320 | 0.612 | — |
|  | Comprehensive × Higher Edu | -0.088 (0.263) | -0.603, 0.426 | 0.737 | — |
| **Service Integration × Higher Edu** | Full × Higher Edu | 0.187 (0.208) | -0.222, 0.595 | 0.370 | — |
| **Cost × Higher Edu** | Cost × Higher Edu | 0.001 (0.006) | -0.010, 0.012 | 0.823 | — |
| **Interaction Method × Higher Income** | Voice × Higher Income | -0.169 (0.248) | -0.655, 0.316 | 0.494 | — |
|  | Video × Higher Income | -0.239 (0.252) | -0.733, 0.255 | 0.343 | — |
| **Service Provider × Higher Income** | AI + Doctor × Higher Income | 0.673 (0.258) | 0.166, 1.179 | 0.009** | — |
|  | Doctor × Higher Income | 0.275 (0.254) | -0.222, 0.772 | 0.278 | — |
| Log-likelihood = -925.634 LR chi² (10) = 122.77  **Note**: ***p < 0.001, **p < 0.01, *p < 0.05. | | | | | |
